# ABPs: diffusible antibacterial toxins secreted by and active against diverse gram-positive bacteria

**DOI:** 10.1128/iai.00297-26

**Published:** 2026-07-22

**Authors:** Jake Colautti, Daniel Mwangi, Joshua J. Woodward, John C. Whitney

**Affiliations:** 1 Department of Biochemistry and Biomedical Sciences, McMaster University 3710, Hamilton, Ontario, Canada; 2 Michael DeGroote Institute for Infectious Disease Research, McMaster University 3710, Hamilton, Ontario, Canada; 3 Department of Microbiology, University of Washington 7284, Seattle, Washington, USA; 4 David Braley Center for Antibiotic Discovery, McMaster University 3710, Hamilton, Ontario, Canada; University of Pittsburgh, Pittsburgh, Pennsylvania, USA

**Keywords:** polymorphic toxins, antibacterial proteins, interbacterial competition, gram-positive bacteria

## Abstract

Competition between organisms is a ubiquitous feature of life on Earth and a major driver of evolutionary innovation. As Earth’s most diverse and abundant organisms, bacteria employ numerous strategies to inhibit the growth of competitors, ranging from the production of diffusible antibiotic metabolites to the secretion of sophisticated protein toxins. Although secreted antibacterial protein toxins have been extensively studied in gram-negative bacteria, analogous systems employed by gram-positive organisms remain comparatively poorly understood. Recent work has shed light on a widespread family of secreted protein toxins associated with co-secreted serine proteases that likely mediate interbacterial competition among gram-positive species. These systems, termed antibacterial protein (ABP) systems, consist of a secreted polymorphic toxin (AbpT), a co-secreted serine protease (AbpP), and a cytoplasmic immunity protein (AbpI). Following proteolytic processing, ABP toxins inhibit the growth of a remarkably diverse range of gram-positive bacteria spanning the phyla Bacillota and Actinomycetota. Existing evidence suggests that these proteins combine the properties of cationic antimicrobial peptides with those of classical polymorphic antibacterial toxins, potentially enabling toxin delivery into distantly related bacteria. In this review, we summarize the current understanding of the organization, mechanisms, and ecology of ABP systems; discuss major outstanding questions regarding toxin import and native biological function; and highlight opportunities for future mechanistic and biotechnological investigation in this emerging field.

## COMPETITION AS A DRIVER OF MOLECULAR INNOVATION

The biological world is the product of billions of years of evolution, shaped by selective pressures acting on genomes and the molecules they encode. Although the precise forces driving the evolution of individual lineages are often difficult to reconstruct, competition between organisms is widely recognized as a major contributor to evolutionary change (1, 2). This evolutionary “Red Queen” dynamic, in which organisms continuously evolve offensive and defensive strategies in response to one another, is thought to contribute substantially to the remarkable molecular and phenotypic diversity observed across the tree of life (3–5). As Earth’s oldest and most abundant organisms, bacteria have participated in these evolutionary conflicts for billions of years and have consequently evolved a vast array of competitive behaviors (4).

The capacity of bacteria to directly inhibit the growth of competitors has been recognized for more than a century, and studies of these antagonistic behaviors have repeatedly revealed fundamental biological insights and enabled important biotechnological innovations. Over a century ago, Gratia identified strains of *Escherichia coli* that produced a molecule capable of inhibiting the growth of other strains, a phenomenon later observed in *Shigella*, *Citrobacter*, and *Klebsiella,* and ultimately attributed to diffusible protein toxins now known as colicins (6, 7). Around the same time, Rogers and Whittier described *Lactococcus* species that inhibited contaminating bacteria in fermentation cultures (8). This activity was subsequently shown to result from the production of the antimicrobial peptide nisin (9). Antimicrobial peptides are now known to be produced by nearly all forms of life, and nisin continues to be widely used as a food preservative and in other industrial applications (10–12). Similarly, the discovery that soil-dwelling bacteria compete through the production of small-molecule antibiotics (13) launched a golden age of antibiotic discovery that ultimately yielded many of the antibiotics used in clinical practice today (14).

Since these early discoveries, our understanding of interbacterial competition has expanded considerably. Bacteria are now known to deploy a diverse array of antagonistic mechanisms, ranging from diffusible molecules such as antibiotics, antimicrobial peptides, and protein toxins to sophisticated molecular machines that directly inject toxic proteins into neighboring cells (15–25). Continued investigation of these systems has revealed unexpected protein functions and biological mechanisms, which have been reviewed extensively elsewhere (4, 26–31). Despite this progress, advances in DNA sequencing, comparative genomics, and protein structure prediction suggest that current knowledge may represent only a small fraction of the competitive mechanisms encoded within the bacterial domain (32).

Much of our current understanding of antibacterial protein toxins derives from studies of competition between gram-negative organisms. Many gram-negative species encode specialized machines that inject toxic proteins into physically adjacent competitor cells (26). Because these systems bypass the need for receptor-mediated uptake, they often exhibit a broad target range that is not constrained by the presence of a specific surface receptor. In contrast, colicins and related families of diffusible antibacterial toxins produced by gram-negative organisms require a specific surface receptor to translocate into target cells and therefore display an extremely narrow range of antibacterial activity typically restricted to a single bacterial species or sub-species lineage (29). Despite these differences in toxin delivery, there are commonalities in the organization of protein toxins across biological systems. Toxic proteins typically contain N-terminal-trafficking domains that enable their secretion from producing organisms or translocation into target cells and C-terminal toxin domains that harbor their lethal activity (32). The evolutionary history of these toxins is readily apparent from their modular, polymorphic organization; C-terminal toxin domains are often associated with diverse N-terminal-trafficking and translocation domains in different species and toxin systems. This modular organization is thought to reflect extensive horizontal transfer and recombination during evolution (32). Antibacterial toxin domains are often enzymatic and generally act by degrading or modifying essential molecules, thus irreversibly perturbing cellular homeostasis and inhibiting target cell growth (32–40). To counteract this activity in the producing cell and prevent the killing of genetically identical kin cells, genes encoding antibacterial toxins are always encoded alongside cognate immunity proteins that typically bind to and inactivate their associated toxins (15, 41). This foundational work in gram-negative organisms has defined the general principles underlying the function of antibacterial protein toxins and provides a framework for understanding these systems in new biological contexts.

**Fig 1:**
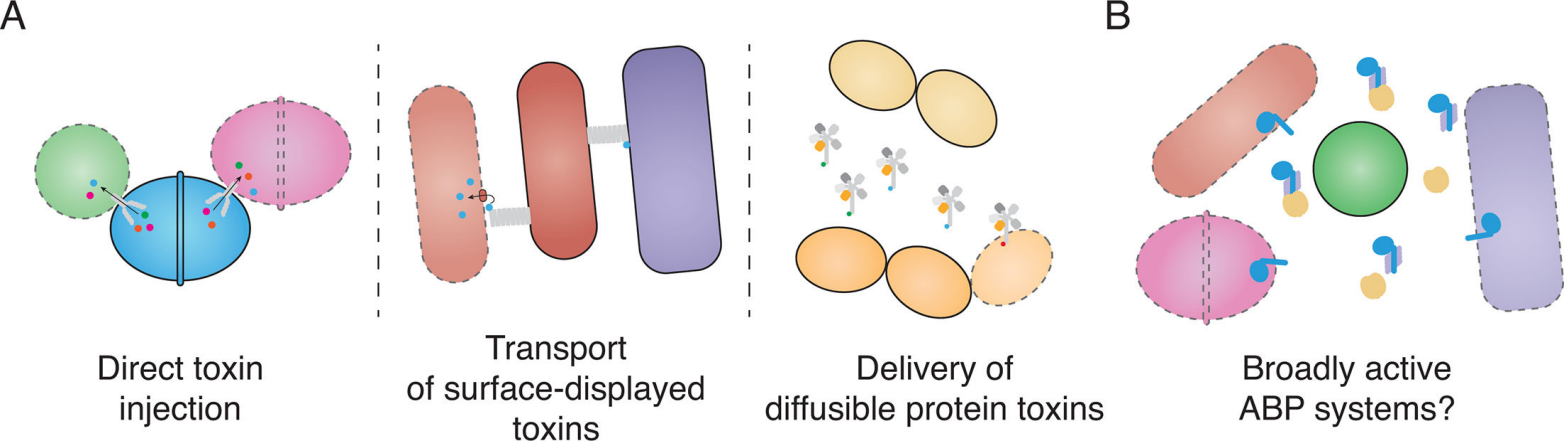
ABP systems challenge the relationship between toxin delivery mechanism and target range. (**A**) Protein toxins are delivered between bacteria by several mechanisms, including direct injection into adjacent cells and receptor-dependent uptake of diffusible or surface-displayed toxins. For diffusible and surface-displayed toxins, interactions with specific receptors on the target cell surface define the range of susceptible organisms. (**B**) In contrast, ABP systems produce diffusible toxins that are active against diverse gram-positive bacteria spanning the phyla Bacillota and Actinomycetota. This broad activity spectrum challenges the prevailing view that diffusible protein toxins require highly specific receptors and are therefore restricted to a narrow range of target organisms. Killed susceptible organisms are depicted with dashed lines.

Gram-positive bacteria inhabit many of the same highly competitive ecosystems as gram-negative organisms and therefore likely engage in extensive interbacterial competition. However, comparatively less is known about antibacterial protein toxins deployed by gram-positive species (Fig. 1A). Several species in the phylum Bacillota export antibacterial toxins using type VII secretion systems (T7SSs), a gram-positive counterpart to the various contact-dependent antagonism systems that mediate competition between gram-negative species (17, 18, 42). Additionally, some gram-positive organisms attach extended filamentous proteins to their cell envelope, delivering the C-terminal toxin domain of these proteins into competitor cells in a manner analogous to that of the gram-negative contact-dependent inhibition (CDI) pathway (21). Recently, *Streptomyces* species were reported to compete with one another by producing diffusible protein complexes termed umbrella toxin particles (25, 43). These complexes recognize surface glycans on competitor *Streptomyces* cells and deliver functionally diverse toxin domains between species within this genus (25, 43). Importantly, these studies have revealed that many of the principles governing protein toxin function in gram-negative bacteria also apply to their counterparts in gram-positive organisms. Toxins that diffuse through the environment or are displayed on the cell surface, such as umbrella toxins and CDI-like toxins, require compatible cellular receptors and therefore act against a very narrow range of competitors (21, 43). Although their translocation mechanisms remain incompletely understood, antibacterial T7SS toxins are thought to be delivered directly into susceptible cells, which may account for their ability to intoxicate phylogenetically diverse competitors (17, 44).

Recent work has identified a widespread family of gram-positive polymorphic toxins that challenge the traditional association between toxin delivery mechanism and target range (Fig. 1B). These systems, termed antibacterial protein systems (ABPs), are encoded by diverse gram-positive bacteria spanning the phyla Bacillota and Actinomycetota and can target a similarly diverse range of species across these phyla (45, 46). Existing evidence suggests that these systems use a mechanism of target-cell entry distinct from that of previously characterized diffusible protein toxins. In this review, we summarize the current understanding of ABPs, discuss major unresolved questions regarding their mechanisms and ecology, and highlight promising avenues for future investigation.

### Organization and function of ABP systems

ABPs were first identified bioinformatically by Li et al., who observed several distinct C-terminal toxin domains associated with a conserved N-terminal domain of unknown function (46). Because this modular organization is characteristic of polymorphic toxins, Li et al. hypothesized that the conserved N-terminal domain serves as a trafficking or translocation module and defines a previously unrecognized toxin family, later termed antibacterial protein toxins (AbpTs) (45, 46). Li et al. subsequently performed a comprehensive analysis of proteins containing this domain, which they named the BetaH domain because it is predicted to adopt an antiparallel beta-sheet surrounding a central alpha-helix (46).

This bioinformatic analysis revealed several important features of AbpT proteins. In addition to their N-terminal BetaH domains, these proteins contain polymorphic C-terminal toxin domains, many of which share sequence and predicted structural similarity to antibacterial toxins associated with colicins, CDI systems, and other interbacterial conflict systems (Table 1) (46). Consistent with their predicted antibacterial function, these toxin genes are encoded alongside bona fide immunity proteins, many of which belong to established immunity protein families (46). Li et al. further observed that AbpT proteins are encoded adjacent to a putative serine protease, later named AbpP, and that both proteins contain signal sequences predicted to direct their co-translational export through the general secretory (Sec) pathway (45, 46). Thus, comparative genomics revealed that ABP systems are defined by three core components: a secreted polymorphic toxin (AbpT), a secreted serine protease (AbpP), and a cytoplasmic immunity protein (AbpI).

**TABLE 1:** Toxin domains associated with ABP systems, their predicted structural families, and known biochemical activities (46)

Toxin domain name	Conserved fold/domain	Specific biochemical activity (where known)	Experimental validation
Ribonucleases			
ColD	BECR	Non-specific RNAse	(45)
ColD-like	BECR	Unknown	
Ntox21	BECR	16s ribosomal RNA cleavage	(45)
Ntox21-like	BECR	Unknown	
ColE3	BECR	Unknown	
Ntox35	BECR	tRNA cleavage	(45, 47)
S8-BECR1	BECR	Unknown	
S8-BECR2	BECR	Unknown	
S8-BECR3	BECR	Unknown	
Deoxyribonucleases			
S8-Restriction endonuclease	PD-(D/E)XK superfamily	Non-specific DNAse	(45)
S8-XHH	HNH superfamily	Non-specific DNAse	(45)
Novel toxin domains			
S8 Vicinal oxygen chelate 1	VOC superfamily	Unknown	
S8 Vicinal oxygen chelate 2	VOC superfamily	Unknown	
S8 Frataxin-like	Frataxin	Unknown	
Ntox33	Novel fold	Unknown	

The predicted Sec-dependent export of AbpT and AbpP led to an initial model in which AbpTs function as diffusible antibacterial toxins analogous to colicins (46). Under this model, AbpTs were proposed to diffuse through the extracellular environment and inhibit competitor cell growth in a contact-independent manner, while AbpP was hypothesized to contribute to this process through its proteolytic activity. Consistent with this hypothesis, the BetaH domain was proposed to function as a receptor-binding domain that determines the range of bacteria susceptible to a given ABP system, likely organisms closely related to the producing strain (46). Finally, Li et al. found that ABP systems are distributed across diverse gram-positive bacteria belonging to the phyla Bacillota and Actinomycetota and are present in isolates from both environmental and human-associated sources, suggesting that these systems function in a wide range of ecological contexts.

Many of these bioinformatic hypotheses were later supported by two parallel experimental studies, which together provide the foundation for our current understanding of ABPs (45, 47). As expected, the C-terminal toxin domains of AbpT proteins display antibacterial enzymatic activity, and their cognate AbpI proteins physically bind and inactivate these toxins with high specificity. Because many of these toxin domains belong to established enzyme families, their activities can often be predicted based on the studies of related proteins. Indeed, several of these predictions have been experimentally validated, revealing distinct AbpT families that target tRNA or rRNA, or function as apparently non-specific ribonucleases or deoxyribonucleases (Table 1) (45).

One of the most distinctive features of AbpT proteins is the phylogenetic breadth of bacteria they can target. Although diffusible toxins, such as colicins and *Streptomyces* umbrella particles, and cell surface-attached CDI toxins display an extremely narrow target range, AbpTs can seemingly inhibit the growth of phylogenetically diverse gram-positive bacteria spanning multiple orders within the phyla Bacillota and Actinomycetota (45). Importantly, all AbpT toxin domains characterized to date act on intracellular targets and are inhibited by cytoplasmic immunity proteins, suggesting that AbpT proteins must enter susceptible cells to exert their lethal activities (45, 46). Previously described diffusible toxins achieve this feat by binding to a cognate receptor on the target cell surface and translocating through a protein translocon. Reliance on a receptor explains the narrow target range of colicins and other diffusible toxins, as subtle variations in surface macromolecules between species or sub-species lineages are often sufficient to disrupt the highly specific interaction between a toxin and its receptor (24, 48). The ability of AbpTs to inhibit such diverse bacteria therefore raises a fundamental question regarding their uptake, as these organisms are unlikely to share a conserved surface receptor capable of mediating toxin entry.

**Fig 2:**
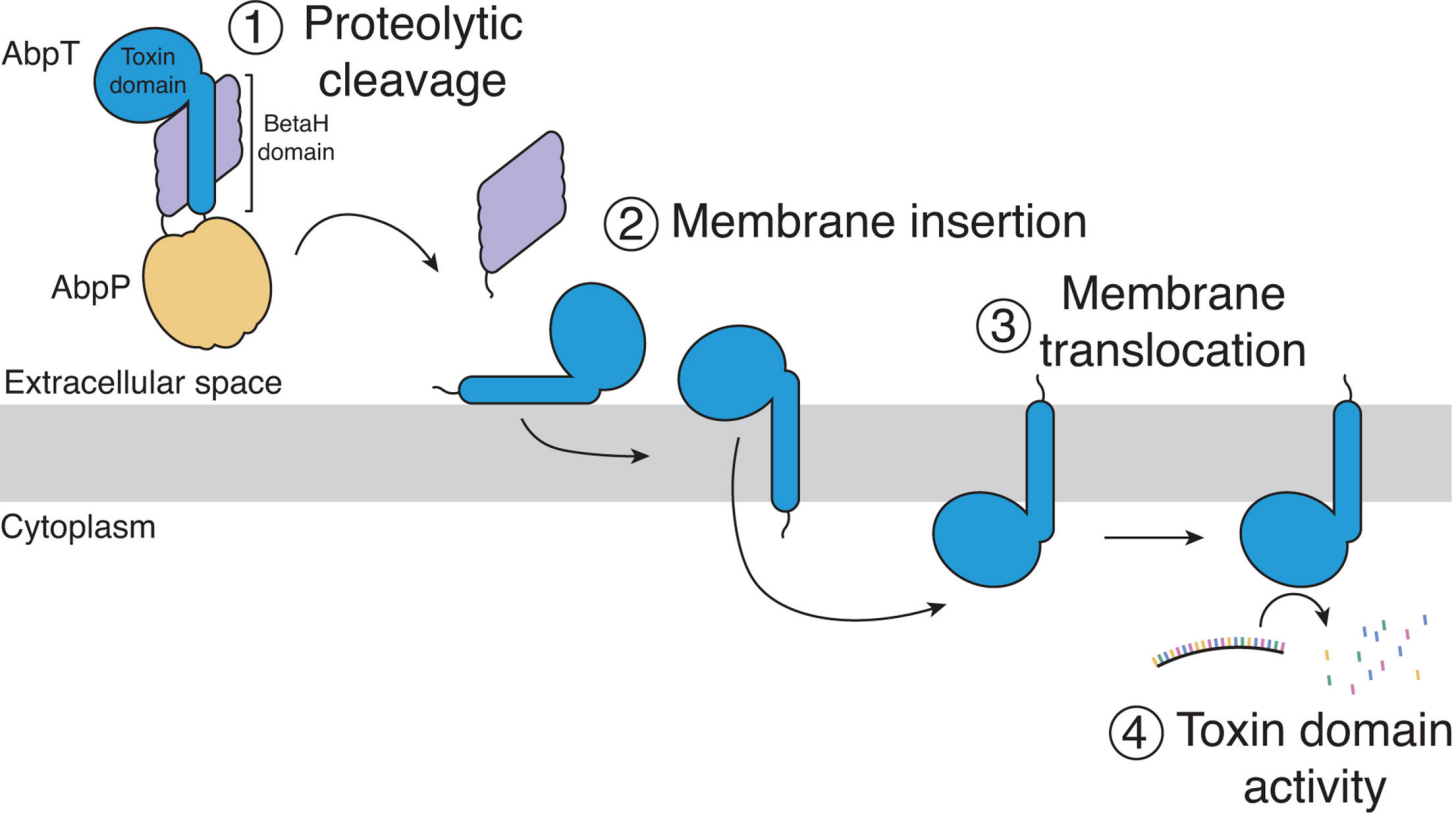
Speculative model of AbpT import into susceptible cells. Following secretion via the general secretory pathway, AbpT proteins are cleaved by their cognate co-secreted AbpP proteases (1). This cleavage event liberates the amphipathic alpha-helix from its surrounding beta-sheet, potentially enabling association with and insertion into the cytoplasmic membrane of a susceptible cell (2). Membrane association may position the highly cationic toxin domain for membrane potential-dependent translocation into the cytoplasm (3). Once in the cytoplasm, the toxin can disrupt essential cellular processes through one of several described enzymatic activities (4).

Although the mechanisms by which AbpTs enter susceptible cells remain enigmatic, existing evidence supports a speculative model for AbpT activation and membrane translocation (Fig. 2). The function of the co-secreted AbpP protease offers one important clue regarding this process. AbpP proteases cleave their cognate toxins within the BetaH domain, releasing two fragments: an N-terminal fragment containing the beta-sheet component of the BetaH domain and a C-terminal fragment containing both the BetaH alpha-helix and the toxin domain (45). This alpha-helix is amphipathic, possessing one hydrophobic face and an opposite, highly cationic face. Such amphipathic helices are characteristic of many cationic antimicrobial peptides (CAMPs), where they facilitate interactions with cellular membranes (45, 49). The observation that AbpP cleavage releases this amphipathic helix from the N-terminal beta-sheet raises the possibility that AbpTs may spontaneously insert into cell membranes in a manner analogous to CAMPs (Fig. 2A). Another important clue regarding AbpT translocation is offered by the AbpT toxin domains themselves, which are highly enriched in basic residues and are therefore extremely positively charged at physiological pH (45). Essentially, all bacteria establish an electrical potential across their cytoplasmic membrane that drives cationic molecules into the cell (50). The membrane potential supplies the energy for active nutrient transport, flagellar motility, and ATP synthesis; maintains the topology of integral membrane proteins; and contributes to the import of several families of antimicrobial peptides and antibiotics (29, 51–56). The observation that AbpT toxin domains are extremely cationic despite their functional diversity suggests that membrane potential may contribute to their translocation across bacterial membranes. Indeed, chemical dissipation of the membrane potential renders diverse bacteria resistant to AbpT activity, suggesting that an active process transports these proteins into susceptible cells (Fig. 2) (45). However, whether membrane potential directly drives spontaneous toxin translocation or instead supports the activity of additional cellular factors that are themselves responsible for AbpT translocation remains unclear.

Despite their broad-spectrum activity, susceptibility to AbpTs varies considerably between species. The minimal inhibitory concentrations of AbpP-cleaved AbpTs differ between organisms, and some gram-positive bacteria are intrinsically resistant to AbpT activity even at protein concentrations in the low micromolar range. These differences in susceptibility likely reflect variation in the electrostatic properties of bacterial cell surfaces. Consistent with this possibility, one study found that AbpT susceptibility was reduced by the expression of enzymes that install cationic modifications on cell-surface macromolecules, such as D-alanylation of wall teichoic acids or lysinylation of membrane phospholipids (47). This finding is consistent with the highly cationic nature of AbpT proteins and suggests that differences in susceptibility arise from electrostatic repulsion between AbpTs and cationic cell-surface modifications. Such modifications have long been known to contribute to resistance against cationic antimicrobial peptides through a similar charge-repulsion mechanism, consistent with a shared mode of membrane interaction (57, 58).

**Fig 3:**
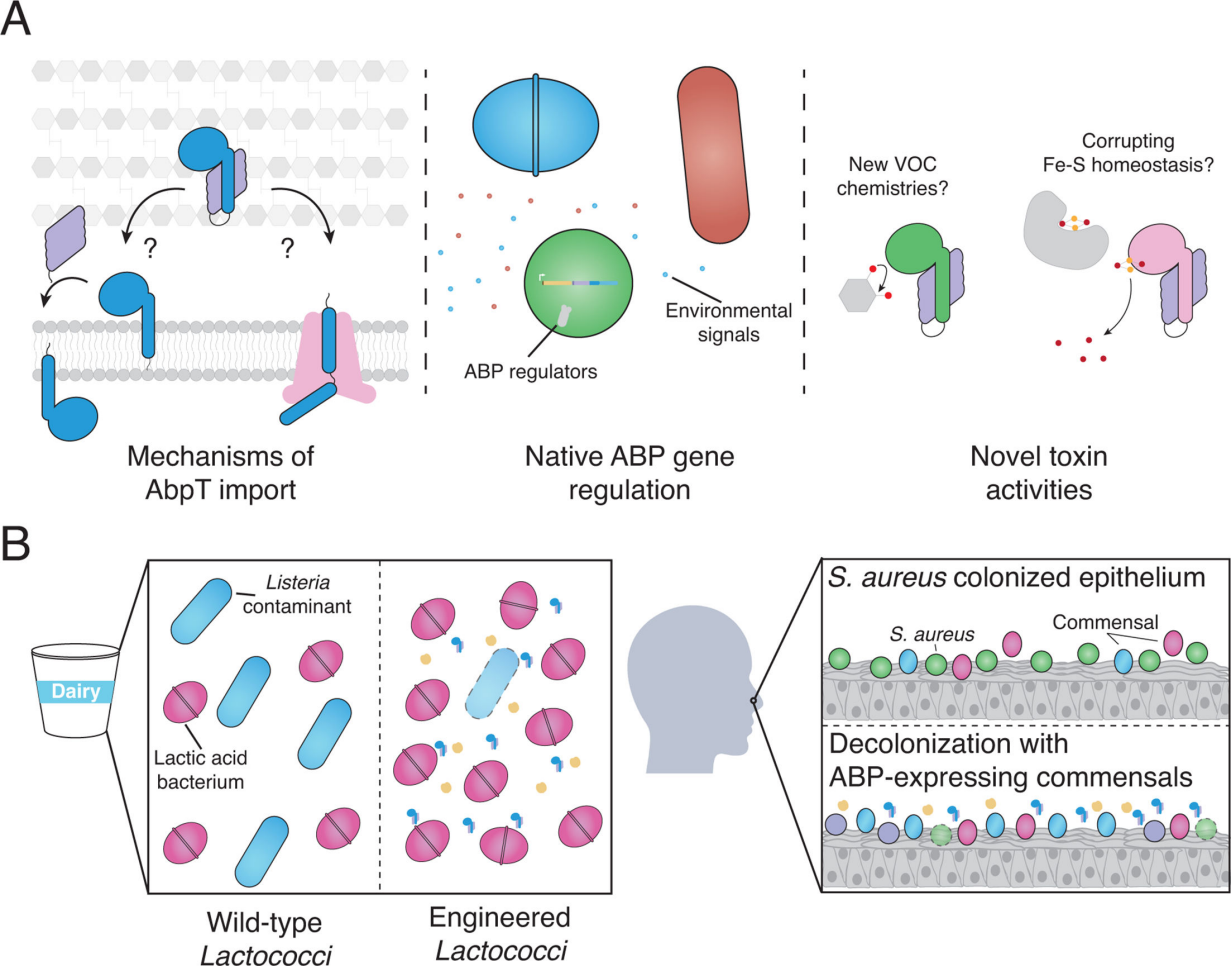
Remaining questions and biotechnological opportunities presented by ABP systems. (**A**) Several important questions remain unresolved, including the mechanisms governing AbpT import, the native signals that control ABP gene expression, and the molecular activities of several unique families of AbpT proteins. (**B**) The broad antibacterial activity of ABP systems presents opportunities to manipulate microbial communities using bacteria engineered to express ABP genes. The use of ABP-expressing lactic acid bacteria to limit *Listeria* contamination of dairy products and the use of engineered commensal *Staphylococcus* or *Corynebacterium* species to reduce colonization by pathogenic bacteria represent two potential applications of ABP systems.

In summary, ABP systems appear to combine the features of cationic antimicrobial peptides with those of classical polymorphic antibacterial toxins. Following proteolytic activation by a cognate secreted protease, AbpT proteins are thought to be translocated across the target cell cytoplasmic membrane in a membrane potential-dependent manner, where their polymorphic toxin domains disrupt cellular homeostasis through diverse mechanisms. While these few existing studies offer a glimpse into how ABPs might function, many important questions remain regarding the biological roles of these systems in their native ecological contexts. Furthermore, the unique properties of ABPs may present opportunities for biotechnological development (Fig. 3).

## CHALLENGES AND OPPORTUNITIES FOR THE FUTURE STUDY OF ABPs

### Mechanisms of membrane translocation

The precise mechanisms governing AbpT import remain among the most important unanswered questions in the field (Fig. 3A). However, several proteins have been reported to spontaneously insert into or translocate across biological membranes, providing a roadmap for future mechanistic investigation of AbpT import. One particularly informative example is the adenylate cyclase toxin CyaA from *Bordetella pertussis. In vitro* studies of isolated membranes revealed that CyaA contains a hydrophobic peptide that spontaneously inserts into lipid bilayers and enables membrane translocation of the adenylate cyclase catalytic domain (59, 60). Similarly, hemolysins and other pore-forming toxins typically contain a membrane-active peptide that inserts into target cell membranes and promotes the formation of ion-conducting pores, often without the involvement of additional proteins in the target cell membrane (61–64). Importantly, much of our understanding of membrane insertion and translocation by these proteins derives from *in vitro* studies examining their behavior in isolated lipid bilayers (65–67). These studies illustrate the power of reconstituted membrane systems for dissecting the molecular determinants of protein insertion and translocation. Similar approaches may therefore prove valuable for determining whether the proposed AMP-like features of AbpTs are sufficient to drive membrane interaction and translocation in the absence of additional cellular factors.

Perhaps the most important question related to AbpT import concerns whether these proteins employ a proteinaceous receptor for entry into susceptible cells, as is the case for all other known families of diffusible antibacterial toxins. Reconstitution of AbpT translocation in isolated lipid bilayers may provide one means of addressing this question. Alternatively, genetic screens that isolate AbpT-resistant mutants provide a complementary strategy for identifying non-essential cellular factors that contribute to AbpT susceptibility. Interestingly, one study that adopted this approach identified the AnrAB translocon of *Listeria monocytogenes* as one factor required for AbpT activity against this organism. This ATP-binding cassette transporter belongs to the Bce transporter family, members of which are widely encoded across the phylum Bacillota and confer resistance to bacitracin and other cationic antimicrobial peptides by transporting them out of the cell membrane (68–70). The finding that inactivation of *anrAB* confers AbpT resistance raises the possibility that Bce transporter activity contributes to the function of ABP systems. However, several lines of evidence suggest that these transporters are unlikely to function as direct AbpT receptors or translocons. A cryo-EM structure of the BceAB transporter from *Bacillus subtilis* revealed that this protein complex lacks a continuous lumen spanning from the extracellular space into the cytoplasm (71). This observation is perhaps unsurprising, given that Bce transporters act on membrane-inserted peptides rather than cytoplasmic substrates, but it raises the question of how AbpTs could translocate into the cytoplasm via these transporters (71). Additionally, sequence conservation among Bce transporters encoded by AbpT-susceptible organisms within the phylum Bacillota is low, and homologs of these proteins are absent altogether in susceptible Actinomycetota such as *Corynebacterium glutamicum* (45). Collectively, these observations argue against a direct role for Bce transporters as AbpT receptors or translocation channels. Instead, Bce transporters may represent genetic determinants of AbpT susceptibility that influence toxin entry indirectly, perhaps by promoting membrane interactions that facilitate AbpT insertion or uptake. Importantly, AbpT import could instead depend on essential and highly conserved membrane proteins, such as the Sec translocon, that would be refractory to identification in conventional genetic screens for toxin resistance. Resolving this question will likely require complementary biochemical, structural, and genetic approaches capable of distinguishing proteins that directly mediate AbpT translocation from factors that influence susceptibility indirectly.

### Native biological functions of ABP systems

Although purified AbpT proteins clearly possess antibacterial activity under laboratory conditions, their native ecological function remains uncertain. Several observations suggest that their biological roles may be more nuanced than simple broad-spectrum antagonism. AbpT proteins generally act at nanomolar concentrations that may be difficult to achieve in many natural environments. Even if these concentrations could be achieved, producing vast quantities of AbpT and AbpP proteins would likely impose a substantial metabolic burden on ABP-secreting organisms. The most comparable protein toxins, colicins, are orders of magnitude more potent than cleaved AbpT proteins and can act at concentrations as low as a single colicin molecule per target cell (29). Additionally, as discussed above, resistance mutations can arise relatively readily through disruption of non-essential cellular pathways. Interestingly, disruption of AnrAB also sensitizes cells to bacitracin and related antimicrobial peptides, raising the possibility that resistance to AbpT intoxication may incur collateral susceptibility to other membrane-active stresses (47). Such fitness trade-offs could constrain the evolution of resistance in natural microbial communities, particularly in environments where organisms are simultaneously exposed to multiple antibacterial compounds.

If AbpT toxins do not utilize cellular receptors in their native context, resistance mechanisms based on altered membrane properties or other general determinants of susceptibility would likely affect multiple ABP systems simultaneously rather than a single toxin family. In contrast to receptor-mediated toxin systems, where toxin and receptor variants can co-evolve through reciprocal selective pressures, such broad resistance mechanisms may reduce opportunities for gene-specific evolutionary adaptation. Collectively, these observations raise the possibility that the remarkable broad-spectrum activity observed under laboratory conditions may not fully reflect the native ecological function of ABP systems. One possibility is that target-cell receptors or other susceptibility-determining factors enhance toxin activity against particular organisms, thereby permitting effective antagonism at concentrations substantially lower than those required in laboratory assays.

On the other hand, there is ample precedent for bacteria to produce broadly antibacterial molecules that function in interbacterial competition. Antimicrobial peptides and broad-spectrum natural product antibiotics represent two of the best-studied examples of this type of antagonism. Although their ecological roles likely extend beyond inhibition of competitor growth, compelling evidence suggests that at least some natural product antibiotics kill a wide range of bacteria in the environment and thereby increase the competitive fitness of the organisms that produce them (72–74). Although the metabolic costs of natural product antibiotic production and protein toxin secretion are difficult to compare directly, both processes likely impose a substantial burden on the producing organism that must be offset by the competitive advantages they confer (75, 76). Moreover, like several species that produce natural product antibiotics, many ABP-encoding organisms inhabit structured ecosystems where modest levels of AbpT secretion into the local environment could achieve high local concentrations of these toxins, thereby conferring a substantial fitness advantage against nearby competitors (46, 77, 78). Furthermore, mathematical modeling studies suggest that although the production of a broad-spectrum toxin is deleterious when the producing organism is a minority member of the community, such toxins confer substantial fitness advantages when the producing organism is present at high densities (79). Therefore, defining the ecological contexts in which ABP systems are naturally expressed will likely be essential for understanding their biological function (Fig. 3A).

A prerequisite for defining these ecological contexts is understanding the signals that control ABP expression. A major obstacle to understanding the native function of ABP systems is that ABP genes do not appear to be constitutively expressed under standard laboratory growth conditions, at least in the model organisms examined to date. Many families of antibacterial toxins are induced in response to stress or danger sensing, a regulatory strategy that enables bacteria to avoid the metabolic costs of toxin production under non-competitive circumstances (80–82). Understanding the native signals that control ABP gene expression could therefore provide important clues regarding how these systems are deployed during competition. One approach is to focus on a single ABP-encoding strain and identify conditions that activate expression of the corresponding gene cluster. Previously described strategies include screening chemical libraries for compounds that induce an ABP reporter or performing genetic screens to identify native regulators of ABP expression (83, 84). A complementary strategy is to adopt a strain-agnostic perspective and search for ecological contexts that activate ABP expression across diverse organisms. For example, large collections of ABP-encoding isolates could be screened against competitors that these species are likely to encounter in their natural environments to identify competitive interactions that trigger ABP production. However, ABP gene clusters are encoded by a small subset of sequenced bacterial genomes, which may limit the assembly of a panel of culturable ABP-encoding isolates for this type of screening approach (46). Furthermore, because the signals and regulatory inputs governing ABP expression likely differ among organisms according to their ecological niches and competitors, both organism-focused and ecology-focused approaches will likely be required to understand the biology of ABP systems.

The widespread distribution of ABP systems among gram-positive bacteria, together with their broad activity spectrum, suggests that these systems may contribute to the structure of microbial communities. One context in which ABP systems may be especially relevant is the human skin microbiome, a highly competitive ecosystem often dominated by *Staphylococcus* and *Corynebacterium* species, both of which are known to produce and be susceptible to AbpT proteins (85, 86). Interbacterial competition within and between these genera has been studied extensively, revealing several antibacterial molecules that prevent colonization by invading species and contribute to the long-term stability of the adult skin microbiome (87–91). The finding that ABP systems are both encoded by and active against *Staphylococcus* and *Corynebacterium* species suggests that these systems may similarly contribute to colonization resistance and microbiome stability (45, 46). Furthermore, because ABP systems deploy functionally diverse toxins against a wide range of target organisms, they may enable human pathogens to outcompete protective commensal species. Although ABP systems are distributed across diverse bacterial lineages, they are present in only a minority of sequenced genomes. As a result, a colonizing pathogen may gain a competitive advantage over resident commensals that do not encode cognate immunity proteins and are therefore susceptible to ABP intoxication.

Similar considerations may apply outside host-associated microbiomes. ABP systems are also widely distributed among plant-associated bacteria and may therefore contribute to the composition and stability of rhizosphere-associated microbial communities (46). A deeper understanding of the roles ABP systems play in these ecological contexts will help define their native biological functions and clarify their contributions to the assembly and stability of microbial communities.

### Novel toxin activities

Although most AbpT proteins contain toxin domains with known or readily inferred functions (Table 1), comparative genomic analyses have identified several toxin domains that appear unique to ABP systems (Fig. 3A) (46). Interestingly, some of these domains share predicted structural similarity with proteins that perform established roles in bacterial physiology but have not previously been implicated in interbacterial antagonism. This observation raises the possibility that cellular proteins involved in homeostasis have been repeatedly co-opted during evolution to function as antibacterial toxins. Characterizing these proteins may therefore reveal previously unrecognized vulnerabilities in bacterial physiology and uncover new mechanisms by which antibacterial toxins inhibit growth.

Two unique families of AbpT proteins contain toxin domains with structural similarity to vicinal oxygen chelate (VOC) enzymes (46). This metalloenzyme family is defined by a common fold and a conserved catalytic mechanism in which a divalent metal cofactor coordinates a substrate or catalytic intermediate through a vicinal oxygen (92). Perhaps owing to the wide range of chemistries enabled by this mechanism, VOC enzymes catalyze diverse biochemical reactions and participate in numerous cellular processes across the tree of life (92). The observation that some AbpT proteins contain a VOC-like C-terminal toxin domain suggests that this versatile enzyme scaffold can be repurposed to function as an antibacterial toxin. However, the precise reactions catalyzed by these proteins are difficult to predict from existing sequence and structural information alone. Unbiased biochemical and metabolomic approaches will likely be necessary to determine how VOC-like AbpT proteins disrupt bacterial homeostasis. Nevertheless, the remarkable functional diversity of VOC enzymes raises the possibility that these toxins operate through mechanisms distinct from those of previously characterized antibacterial proteins, potentially revealing new biochemical activities and vulnerabilities in bacterial physiology.

Another AbpT family contains a toxin domain with structural similarity to the frataxin protein family (46). Although their precise molecular activities remain controversial, frataxin proteins play integral roles in iron homeostasis and iron-sulfur (Fe-S) cluster assembly in organisms ranging from bacteria to humans (93, 94). It has therefore been hypothesized that frataxin-like AbpT proteins interfere with the Fe-S cluster assembly in target bacteria, disrupting cellular homeostasis in one of several conceivable ways. By inhibiting Fe-S cluster biogenesis, these toxins could impair respiratory chain function and cellular energy production (95). Alternatively, they may act more indirectly by perturbing iron homeostasis and promoting the accumulation of redox-active iron that initiates oxidative damage (96, 97). Together, these observations highlight the potential of ABP systems to reveal previously unrecognized mechanisms of bacterial antagonism and expand our understanding of the cellular processes that can be exploited during interbacterial conflict.

### Opportunities for biotechnological development

The capacity of ABP systems to inhibit the growth of diverse gram-positive species presents several potential opportunities for biotechnological development (Fig. 3B). Because ABP systems are encoded by only three genes and rely on the highly conserved general Sec pathway for export from the producing organism, they should be readily transferable between bacterial species. In principle, heterologous expression of an ABP system could provide a competitive advantage against susceptible organisms (46). Such an approach could circumvent the need to purify AbpP and AbpT proteins and may provide a comparatively simple means of altering microbial community composition in favor of desirable species. For instance, several described AbpT proteins display potent antimicrobial activity against *L. monocytogenes*, the causative agent of listeriosis, a severe and sometimes deadly gastrointestinal disease often associated with the contamination of preserved or fermented dairy products (98). In a strategy comparable to those employed with other bacteriocin-producing organisms, lactic acid bacteria used in the production of these foods could be engineered to heterologously express an ABP system as a potential means of limiting *L. monocytogenes* contamination (99–101).

A similar but more ambitious goal may be to engineer an AbpT-producing probiotic *Staphylococcus* or *Corynebacterium* species that can be used to eliminate colonization by pathogenic *Staphylococcus aureus*. Because a significant fraction of *S. aureus* infections originate from colonization of the nasal mucosa and other body surfaces, strategies that decolonize *S. aureus* from these sites have received considerable attention (102, 103). Indeed, recent work has identified commensal *Staphylococcus* species that produce natural product antibiotics and bacteriocins and have shown promise in reducing *S. aureus* colonization in human clinical trials, demonstrating the feasibility of commensal-based decolonization strategies (87, 104). While ABP systems alone are likely insufficient to clear *S. aureus* colonization, their simple genetic organization may make them attractive components of engineered commensal strains. Importantly, AbpT proteins display limited activity against wild-type *S. aureus* due to the presence of cationic moieties on the cell surface of this organism. One possibility would be to combine ABP systems with additional co-secreted enzymes that remove these modifications, thereby increasing the susceptibility of *S. aureus* to AbpT intoxication. However, gaps in our understanding of ABP biology, including an incomplete picture of toxin import, variability in susceptibility across and within species, and the prevalence and significance of intrinsic resistance mechanisms, complicate the efforts to rationally deploy these systems therapeutically. A deeper mechanistic understanding of ABP systems will therefore be essential before they can be effectively harnessed for therapeutic applications.

## CONCLUDING REMARKS

Recent bioinformatic, genetic, and biochemical studies have revealed several features that make ABPs unique. These systems appear to combine the properties of cationic antimicrobial peptides with those of polymorphic toxins and are capable of inhibiting a wide range of gram-positive species through diverse enzymatic mechanisms (45, 47). Despite this rapid progress, many important questions remain unanswered. Among the most important are the mechanisms governing AbpT import, the regulation and ecological functions of ABP systems, and the activities of their unique toxin domains. Finally, the finding that a simple three-gene cluster can encode proteins with such a surprising spectrum of antibacterial activity raises the possibility that other broadly acting diffusible antimicrobial proteins remain to be discovered. As is often the case in studies of interbacterial competition, answering these questions will likely reveal unexpected biology and further expand our understanding of the diverse mechanisms bacteria use to antagonize their competitors.
